# CuGaS_2_ and CuGaS_2_–ZnS Porous Layers from Solution-Processed Nanocrystals

**DOI:** 10.3390/nano8040220

**Published:** 2018-04-05

**Authors:** Taisiia Berestok, Pablo Guardia, Sònia Estradé, Jordi Llorca, Francesca Peiró, Andreu Cabot, Stephanie L. Brock

**Affiliations:** 1 Catalonia Institute for Energy Research—IREC, Sant Adrià de Besòs, 08930 Barcelona, Spain; taisiia.berestok@gmail.com (T.B.); pavyel1980@gmail.com (P.G.); 2 LENS-MIND, Departament d’Enginyeries i Electrònica i Institut de Nanociència i Nanotecnologia (In2UB), Universitat de Barcelona, 08028 Barcelona, Spain; sestrade@ub.edu (S.E.); francesca.peiro@ub.edu (F.P.); 3 Institute of Energy Technologies, Department of Chemical Engineering and Barcelona Research Center in Multiscale Science and Engineering. Universitat Politècnica de Catalunya, EEBE, Eduard Maristany 16, 08019 Barcelona, Spain; jordi.llorca@upc.edu; 4 ICREA, 08010 Barcelona, Spain; 5 Department of Chemistry, Wayne State University, Detroit, MI 48202, USA

**Keywords:** aerogel, xerogel, porous layer, CuGaS_2_/ZnS, photoresponse, nanomaterial

## Abstract

The manufacturing of semiconducting films using solution-based approaches is considered a low cost alternative to vacuum-based thin film deposition strategies. An additional advantage of solution processing methods is the possibility to control the layer nano/microstructure. Here, we detail the production of mesoporous CuGaS_2_ (CGS) and ZnS layers from spin-coating and subsequent cross-linking through chalcogen-chalcogen bonds of properly functionalized nanocrystals (NCs). We further produce NC-based porous CGS/ZnS bilayers and NC-based CGS–ZnS composite layers using the same strategy. Photoelectrochemical measurements are used to demonstrate the efficacy of porous layers, and particularly the CGS/ZnS bilayers, for improved current densities and photoresponses relative to denser films deposited from as-produced NCs.

## 1. Introduction

The solution-based processing of semiconductor films has a number of advantages over the use of vacuum-based technologies. Solution-based processes require lower capital investments, have associated lower maintenance costs, and provide higher production throughput and material yields. These characteristics make them highly appropriate for large scale industrial production. Among the different solution-processing technologies, the deposition of inks formulated from nanocrystals (NCs) is particularly interesting as it allows unparalleled control over material properties and layer nano/microstructure, and it provides crystalline layers without mediating a thermal annealing step, thus reducing processing costs. However, in the absence of a sintering step, ink-based processes generally result in layers characterized by poor electrical conductivities, which is a drawback in most applications. While a thermal annealing is frequently used to improve performance, such treatment spoils the main advantages of NC-based solution processes, such as the precise composition control and the cost reduction associated with the production of crystalline layers without the need of a sintering process. Additionally, even annealed NC-based layers contain significant amounts of carbon coming from added binders and from the surface ligands used to control NC growth and render NCs soluble in the ink media [1]. To fully remove carbon, heat treatments in an oxygen atmosphere are needed, but this is not compatible with materials that are susceptible to oxidation, such as chalcogenides. An alternative strategy to remove organics is the use of solution-based treatments, but these processes often involve toxic compounds such as hydrazine [2,3]. Besides toxicity, if not properly controlled, such solution-based ligand-stripping strategies can result in large concentrations of surface traps that may strongly limit the material performance [4]. An alternative strategy to remove organic ligands from NC-based layers and cross-link the NCs to facilitate charge transport involves using a non-oxygen-transferring oxidant [5,6,7]. This oxidizing agent produces chalcogen-chalcogen bonds between the NCs, potentially reducing surface recombination sites and facilitating charge transfer between NCs [8,9]. The concentration of this oxidizing agent also allows the porosity of the final material to be controlled. In this regard, in the particular case of photocatalysis and photoelectrocatalytic applications, the formation of porous layers may be advantageous since porous materials allow penetration of reactive species and expose huge surface areas for interaction with the media [10].

Relative to metal oxides, metal sulfide NCs are of great importance because of their covalent bonding, which results in higher charge carrier mobilities, broader bands and narrower energy gaps [1]. Relative to other selenides and tellurides, sulfides present an obvious advantage in terms of abundance and cost. Among metal sulfides, CuGaS_2_ (CGS), a p-type semiconductor, has gained special attention due to its relatively good stability, moderate cost and toxicity and its direct band gap in the visible (2.4 eV) [11]. CGS is employed in green-light emitting LEDs as well as in visible-light-induced photocatalysis. Furthermore, its relatively large band gap makes it promising as host material for the introduction of intermediate band states to widen its absorption spectra [12,13].

In the present work, we use the oxidative assembly strategy [14] to produce porous CGS NC-based layers. We further extend this strategy to the production of porous multilayers of CGS and ZnS, and of porous composite layers combining CGS and ZnS NCs. We additionally characterize their photoelectrochemical performance toward hydrogen evolution from a Na_2_SO_4_-containing water solution.

## 2. Materials and Methods

### 2.1. Materials

Copper(II) acetylacetonate (Cu(acac)_2_, 98%), gallium(III) acetylacetonate (Ga(acac)_3_, 99.99%), trioctylphosphine oxide (TOPO, 99%), zinc chloride (ZnCl_2_, ≥98%), oleylamine (OAm, 70%), sulfur powder (99.998%), thioglycolic acid (TGA, ≥98%), 11-mercaptoundecanoic acid (MUA, 95%), tetramethylammonium hydroxide pentahydrate (TMAOH, ≥97%), dodecanethiol (DDT, ≥98%), tert-dodecanethiol (t-DDT, 98.5%), tetranitromethane (TNM, 95%), sodium sulfate (Na_2_SO_4_, ≥99%) and potassium chloride (KCl, ≥99%) were purchased from Sigma-Aldrich (Madrid, Spain). Hexane, methanol and acetone were of analytical grade and were purchased from Panreac (Barcelona, Spain). Glass substrates coated with fluorine doped tin oxide (FTO, ~8 Ω/sq) were acquired from VWR (Leuven, Belgium). All the syntheses were carried out using standard air-free Schlenk-line techniques.

### 2.2. Synthesis of CGS NCs

For the experiment, 1 mmol of Cu(acac)_2_ and 1 mmol of Ga(acac)_3_ together with 3.5 mmol of TOPO were mixed with 10 mL of OAm upon magnetic stirring. After degassing at 90 °C for 60 min under vacuum, an argon atmosphere was introduced and the reaction mixture was heated to 270 °C. At 150 °C, 1.12 mmol (0.25 mL) of DDT and 7.4 mmol (1.75 mL) of t-DDT were injected, which changed the color of the solution from dark blue to clear yellow. While increasing temperature, the solution color further changed to clear brown, indicating the NC nucleation, and dark-brown at 250 °C. The mixture was allowed to react at 270 °C for 30 min and afterwards the heating mantle was removed to allow the solution to cool down naturally. NCs were isolated by adding 5 mL of acetone and centrifuging at 5700 rpm for 5 min. The supernatant was discarded and the precipitate was redispersed in 5 mL of hexane. Additional purification steps were performed following the same procedure. Finally, NCs were redispersed in 5 mL of hexane for later use.

### 2.3. Synthesis of ZnS NCs

The experiment also involved 2 mmol of ZnCl_2_ and 6.5 mmol of TOPO being dissolved in 10 mL of OAm under Ar atmosphere at 170 °C for 60 min. The clear transparent solution formed was allowed to cool down to room temperature. At this point, a degassed mixture of 2.5 mmol of sulfur in 5 mL of OAm was injected. Then, the reaction mixture was heated to 320 °C and allowed to react at this temperature for 60 min. Afterwards, the heating mantle was removed to allow the solution to cool down naturally and NCs were collected and washed by adding 5 mL of ethanol followed by centrifugation. The washing procedure was repeated at least 2 more times and NCs were finally dispersed in 5 mL of hexane for later use.

### 2.4. TGA Ligand Exchange

In addition, 20 mg of CGS NCs were dispersed through 15 min of sonication and shaking in 10 mL of methanol containing 4 mmol of TGA and the proper amount of TMAOH to adjust the pH to 10. Afterwards, NCs were washed by adding 10 mL of acetone and centrifugation at 5700 rpm for 5 min. The purification step was repeated twice, followed by redispersion of NCs in 1 mL of methanol.

### 2.5. MUA Ligand Exchange

1 mL of a ZnS NCs solution (20 mg/mL in hexane) was mixed with 1 mL of a MUA solution (4 mmol in 10 mL of methanol) at pH 10 (adjusted using TMAOH). The resulting bi-phase solution was shaken and sonicated for 15 min. Afterwards, the upper part was removed and 5 mL of fresh acetone was added. This step was followed by centrifugation for 5 min at 5000 rpm. The obtained precipitate was redispersed in 1 mL of methanol for later use.

### 2.6. NCs Films

1 mL of a hexane or methanol solution of NCs (20 mg/mL) was spin-coated on FTO-coated glass substrates at a rotation speed of 1500 rpm for 20 s. Afterwards, the NC layer was annealed at 250 °C for 60 min under argon flow.

### 2.7. Porous Xerogel Films

For the experiment, 1 mL of a methanol solution of TGA-capped CGS NCs or MUA-capped ZnS NCs (20 mg/mL) were spin-coated on FTO-coated glass substrates at a rotation speed of 1500 rpm for 20 s. Immediately afterwards, the NC layer was dipped for 1 min into an acetone solution containing 3 vol % of TNM. Films were rinsed with fresh methanol to remove by-products and afterwards annealed at 250 °C for 60 min under argon flow.

### 2.8. Gel Preparation

Fifty microliters of a TNM solution (3 vol % in acetone) was added into a 2 mL methanol solution containing TGA-capped CGS NCs (10 mg/mL). The mixture was shaken vigorously for 30 s and kept undisturbed for the whole gelation process. The gelation process visually evolved for 15 min after the addition of the TNM solution, but it was left to react for two days to ensure completion. Then, the solvent was exchanged with fresh methanol or acetone to remove TNM-residues and by-products, in 1–2 h steps over 2 days. This process must be carried out with special care in order to not damage the porous network of the wet-gel. The wet-gel could be dried into a xerogel at room temperature and ambient pressure, resulting in a significant shrinkage.

### 2.9. Aerogel Preparation

The wet-gel (in acetone) was loaded in a supercritical point drier chamber, which was filled with liquid CO_2_ and kept filled overnight. Then, the chamber was half drained and filled with fresh liquid CO_2_. This procedure was repeated at least 6 times in one-hour intervals in order to completely replace acetone by liquid CO_2_. Then, the chamber was completely filled with liquid CO_2_ and heated above 39 °C. Upon heating, pressure increased up to 75–80 bars, thus surpassing the supercritical point of CO_2_. The sample was kept under these conditions for 1 h followed by releasing the pressure at constant temperature.

### 2.10. Photoelectrochemical Measurements

Photocurrent measurements were performed using a three-electrode cell configuration with a Pt-mesh as a counter electrode (2 cm^2^ surface area) and a Ag/AgCl reference electrode filled with 3 M KCl solution. A 0.1 M aqueous solution of Na_2_SO_4_ at pH = 7 was used as electrolyte. The bias voltage was applied to the working electrode through an electrical contact to the uncoated part of the FTO layer. 1 cm^2^ of the tested semiconductor layer was in contact with the electrolyte. Illumination was provided by 8 xenon lamps (35 W each, Osram, Madrid, Spain) radially distributed with a total power of 280 W and irradiance on the sample of ca. 100 mW/cm^2^.

### 2.11. Characterization

Transmission electron microscopy (TEM) characterization was carried out using a ZEISS LIBRA 120 (Carl Zeiss, Jena, Germany), operating at 120 kV. Samples were prepared by drop casting a diluted NC solution onto a carbon-coated copper grid (200 mesh). Scanning electron microscopy (SEM) analysis was carried out using a ZEISS Auriga microscope (Carl Zeiss, Jena, Germany). For SEM characterization, NCs dispersed in proper solvent were drop casted onto silicon substrates. X-ray power diffraction (XRD) analyses were carried out on a Bruker AXS D8 ADVANCE X-ray diffractometer (Bruker, Karlsruhe, Germany) with Ni-filtered (2 µm thickness) Cu Kα1 radiation (λ = 1.5406 Å). Samples were drop casted (200–500 µL at a concentration of about 3 mg/mL) onto a zero-signal silicon wafer. UV-Vis absorption spectra were recorded on a PerkinElmer LAMBDA 950 UV-Vis spectrophotometer (PerkinElmer, Walthham, MA, USA). Samples were prepared by diluting 100 μL in 2 mL of hexane inside a quartz cuvette with a 10 mm path length. Fourier Transform Infrared (FTIR) spectroscopy investigations were carried out using a PerkinElmer FT-IR 2000 spectrophotometer (PerkinElmer, Walthham, MA, USA). Spectra were recorded from 500 to 4000 cm^−1^. Samples were characterized by electrochemical impedance spectroscopy (EIS) using a versaSTAT3 (Ametek, Madrid, Spain). Measurements were conducted in the frequency range from 100 kHz to 1 mHz with a 5 mV AcCamplitude using the three-electrode cell configuration with the same conditions used for photocurrent measurements. To perform thickness measurements, the FTO substrate was partially masked prior to the spin-coating of the NC inks. The thickness profiles were taken at the edge of the film sample and bare FTO using a Sensofar Plu Neox laser scanning confocal microscope (Sensofar, Terrassa, Spain) with a Nikon TU Plan Fluor objective at a magnification of 100×. X-ray photoelectron spectroscopy (XPS) was done on a SPECS system (SPECS GmbH, Berlin, Germany) equipped with an Al anode XR50 source operating at 150 mW and a Phoibos 150 MCD-9 detector (SPECS GmbH, Berlin, Germany). The pressure in the analysis chamber was kept below 10^−7^ Pa. The area analyzed was about 2 mm × 2 mm. The pass energy of the hemispherical analyzer was set at 25 eV and the energy step was maintained at 1.0 eV. Data processing was performed with the Casa XPS program (version, Casa Software Ltd., Teignmouth, UK). Binding energies were shifted according to the reference C 1s peak that was located at 284.8 eV. Thermogravimetric (TG) analyses were carried out using a PerkinElmer Diamond TG/DTA instrument (PerkinElmer, Walthham, MA, USA). For TG analysis, samples were dried and 20 mg of the dried powder was loaded into a ceramic pan. Measurements were carried out in an Ar atmosphere from ambient temperature to 500 °C at a heating rate of 2 °C/min.

## 3. Results

**Figure 1:**
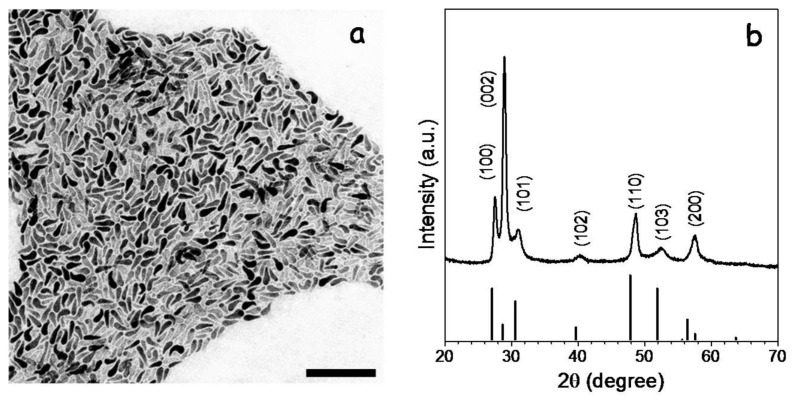
Representative TEM micrograph (**a**) and XRD pattern (**b**) of CGS NCs with wurtzite crystal phase. TEM scale bar = 200 nm. The JCPDS (Joint Committee on Powder Diffraction Standards) 001-1280 phase standard card, corresponding to wurtzite CGS, is included as a reference [16].

CGS NCs were synthesized using a previously reported procedure with some modifications (see experimental section for details) [15]. Briefly, NCs were produced through the reaction of DDT and t-DDT with Cu(acac)_2_ and Ga(acac)_3_ dissolved in OAm and in the presence of TOPO. The reaction mixture was heated up to 270 °C and maintained at this temperature for 30 min. From this procedure, CGS NCs with the wurtzite crystal phase, tadpole geometry and an average length of ca. 50 nm were produced (Figure 1).

**Figure 2:**
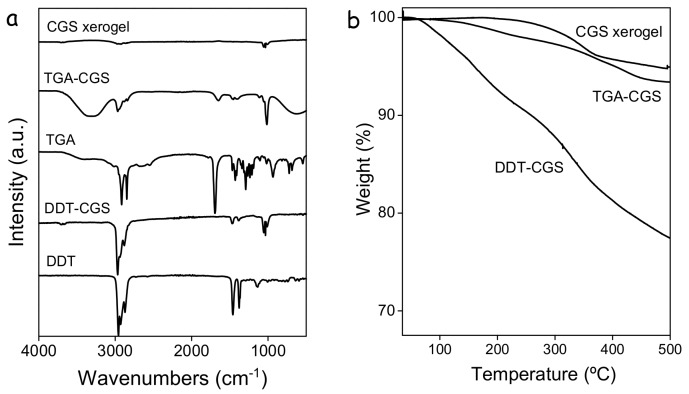
(**a**) from bottom to top: FTIR spectra of DDT; as-prepared CGS NCs (DDT-CGS); TGA; NCs functionalized with TGA (TGA-CGS); and the NC xerogel obtained by exposing TGA-NCs to the oxidant solution and naturally drying them; (**b**) thermogravimetric profile of the DDT-CGS NCs, TGA-CGS NCs and CGS xerogel.

FTIR characterization showed the as-prepared CGS NCs to contain significant amounts of organic ligands, as revealed by the presence of peaks at 2924 and 2830 cm^−1^ that correspond to C–H stretching (Figure 2a). This native surface organic ligand, most probably DDT according to previous reports [17], was displaced using TGA. For this purpose, as-prepared CGS NCs (DDT-CGS) were dispersed through sonication and shaking in a methanol solution of TGA and the proper amount of TMAOH to adjust the pH to 10. After purification, FTIR spectra showed a drastic reduction of the C–H peak intensity consistent with the shorter organic chain of TGA. Thermogravimetric analysis also showed a significantly lower decrease of the weight loss from the TGA-CGS NCs compared with the DDT-CGS NCs, consistent with the lower organic content of the former (Figure 2b).

**Figure 3:**
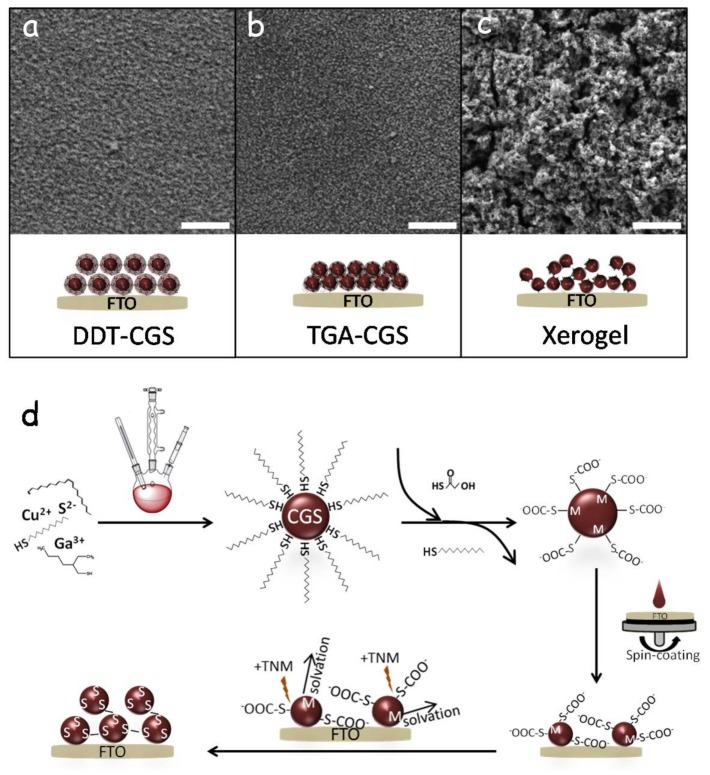
(**a**–**c**) representative SEM micrographs and schematic representations of the CGS layers produced via spin-coating of DDT-CGS (**a**), TGA-CGS (**b**), and TGA-CGS after undergoing oxidative assembly with TNM to form a xerogel film (**c**). Scale bars = 1 μm; (**d**) schematic representation of the procedure to produce porous xerogel NC films by removal of the thiol ligand through a non-oxygen transferring oxidant, TNM.

After ligand exchange, TGA-CGS NCs in methanol were spin-coated onto FTO substrates to form CGS layers. Subsequently, TGA was removed using a TNM solution in acetone. For this purpose, immediately after spin coating, CGS layers were dipped into an acetone solution containing 3 vol % TNM for 1 min. Layers were washed afterwards with methanol and allowed to dry naturally. Finally, they were annealed at 250 °C in Ar for 60 min. For comparison, we also produced CGS layers using DDT-CGS and TGA-CGS but with no ligand displacement/oxidation step. Figure 3 shows top-view SEM images of the different layers. The films produced after the displacement of TGA showed a much rougher surface than those obtained from DDT-CGS and from TGA-CGS (without TGA displacement/oxidation) suggesting greater porosity. As schematized in Figure 3d, exposure of TGA-CGS to the non-oxygen transferring oxidizer (TNM) resulted in partial removal of TGA. Unprotected surface metal ions were then solvated to result in a chalcogen rich NC surface that underwent NC-NC cross-linking through oxidation-induced chalcogen-chalcogen bonding. This mode of NCs cross-linking resulted in the formation of a porous network of interconnected NCs in solution—an NC-based gel [18]. The final material still contains TGA on the surface of particles. The ligand-oxidation process is competitive with ZnS sulfide oxidation; as portions of the particle are de-protected, they undergo assembly to form a linked network, but a portion of the particles still remains ligand-capped. To fully remove the ligand-related carbon from the final film, an additional chemical or thermal treatment of the layer is required.

**Figure 4:**
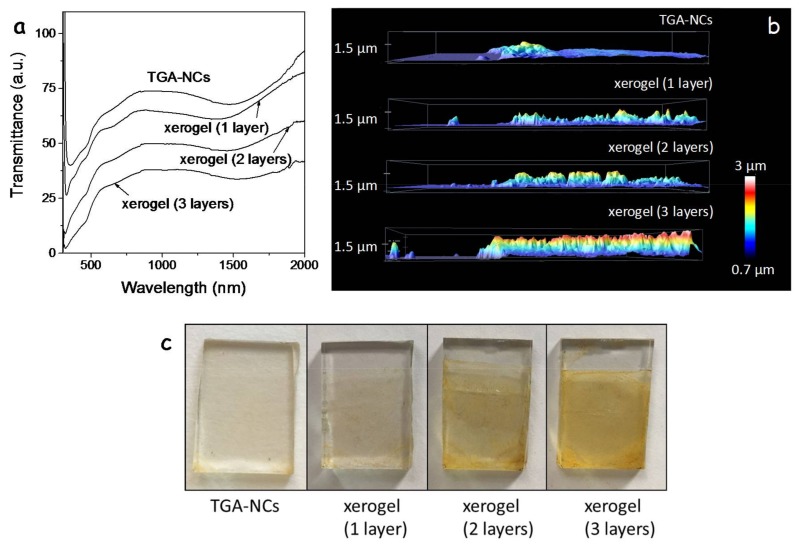
(**a**) transmittance spectra of films produced from as-synthesized NCs, TGA-NCs, and xerogel films with different numbers of layers (1 layer, 2 layers; 3 layers); (**b**) thickness profiles of the produced films; (**c**) optical photographs of the films.

The layer thickness could be controlled through the NC concentration in solution and the number of spin coated layers. Figure 4a shows the UV-Vis spectra of a TGA-CGS NC film and three xerogel films with different thicknesses produced by the successive deposition of one, two or three CGS layers followed by their gelation. Notice how the transmittance of the layers decreases with the film thickness. Transmittance is slightly lower for the single xerogel film compared with the TGA-CGS film, which we associate with the higher scattering of the former. Figure 4b,c display the thickness profiles of the films and their optical photographs. 

**Figure 5:**
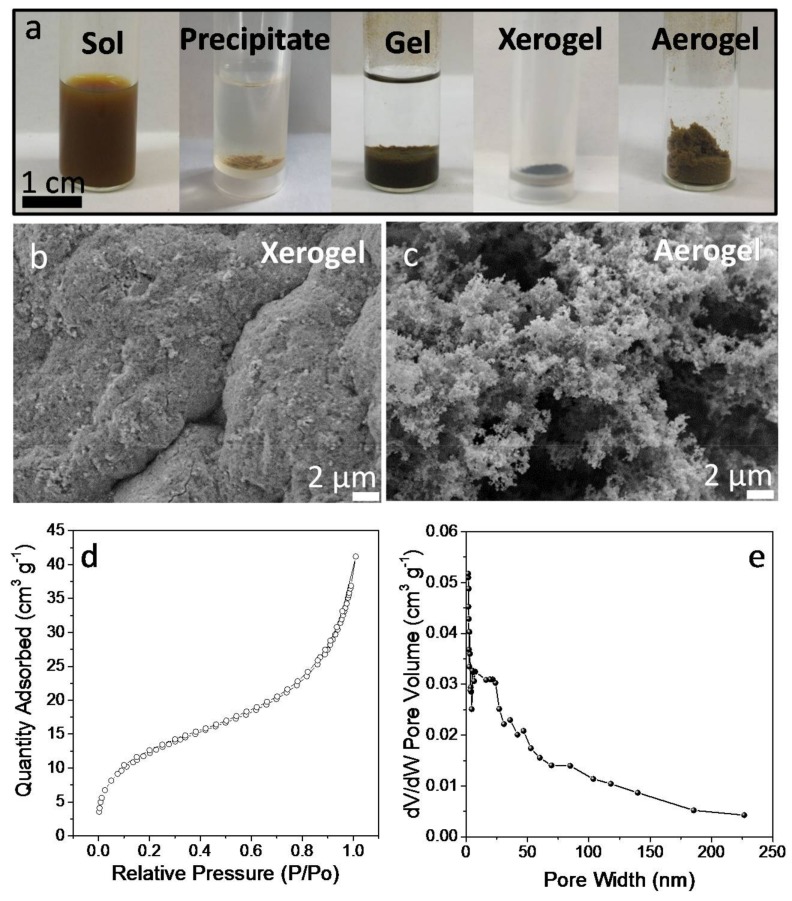
(**a**) optical images of the CGS NC-based sol, precipitate, gel, aerogel and xerogel; (**b**) SEM micrograph of a CGS xerogel; (**c**) SEM micrograph of a CGS aerogel; (**d**) adsorption/desorption isotherm cycle from a CGS NC-aerogel, and (**e**) its corresponding BJH pore size distribution.

To gain insight into the mechanism of displacement of the TGA ligand in TGA-CGS NCs and the NC network formation, the same treatment was applied to unsupported NCs. In this case, the TNM solution in acetone was injected into a colloidal solution of TGA-CGS NCs in methanol. Upon injection, a gel started to form. Gelation visually evolved for 15 min but was allowed to carry on for 48 h. Afterwards, the CGS NC gel was rinsed with fresh methanol and allowed to dry naturally into a xerogel (Figure 5). FTIR characterization (Figure 2a) of the CGS xerogels revealed a significant decrease of the intensity of the C–H stretching peaks when compared with TGA-CGS NCs, providing evidence of a strong, but not complete, reduction of the amount of organics at the CGS NC surface. Thermogravimetric analysis (Figure 2b) further demonstrated the reduction of the organic content.

SEM micrographs of the obtained CGS xerogels displayed a porous structure of interconnected NCs (Figure 5b,c). When drying the CGS gel from supercritical CO_2_ (see experimental section for details), highly porous aerogels, characterized with Brauner-Emmett-Teller (BET) surface areas of 46 m^2^·g^−1^, were obtained. Approximating the CGS NC geometry as cylindrical and considering an average cylinder length of 50 nm and an average diameter of 15 nm, their total surface area would be 64 m^2^·g^−1^. Thus, within this approximation, the produced aerogels were able to keep over 70% of the surface area of the colloidal NCs. CGS aerogels showed type IV adsorption-desorption isotherms with a combination of H1- and H3-type hysteresis loops, consistent with a mesoporous structure (Figure 5d). Barrett–Joynes–Halenda (BJH) plots displayed a broad pore size distribution, characteristic of their aerogel nature (Figure 5e).

**Figure 6:**
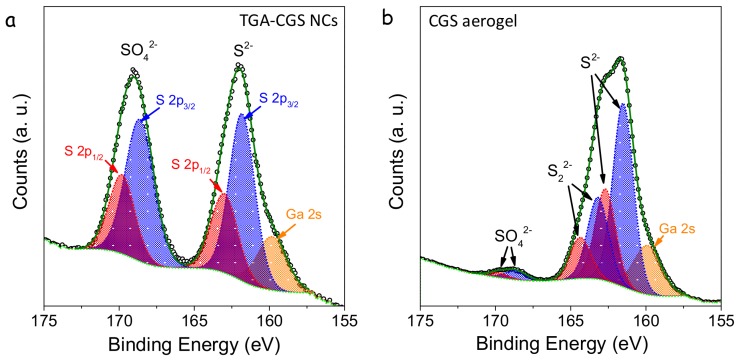
S 2p region of the XPS spectra of the TGA-CGS NCs (**a)** and the CGS NC-based aerogel (**b**). S 2p3/2 states are plotted in blue and S 2p1/2 states in red. In the same region, the Ga 2s state is observed.

XPS analysis confirmed the formation of chalcogen-chalcogen bonds between the particles (Figure 6). XPS spectra of TGA-NCs exposed to air displayed two contributions to the S 2p regions, one associated with the lattice S^2−^ (S 2p_3/2_ binding energy = 161.8 eV) and the second one associated with a sulphate (S 2p_3/2_ binding energy = 168.6 eV) [19]. In the XPS spectra of the TNM-treated sample, a third component became evident. This third chemical state (S 2p_3/2_ binding energy = 163.2 eV) had a slightly lower binding energy than elemental sulfur (S 2p_3/2_ binding energy = 163.7 eV) [19]. Thus, we associated it to sulfur with a chemical environment compatible with that of disulphides, which is consistent with formation of S_2_^2−^ type linkages. Additionally, the sulphate component was strongly decreased in the gelated sample, and the treatment with TNM eliminated both suface thiolates and surface oxidation layers.

**Figure 7:**
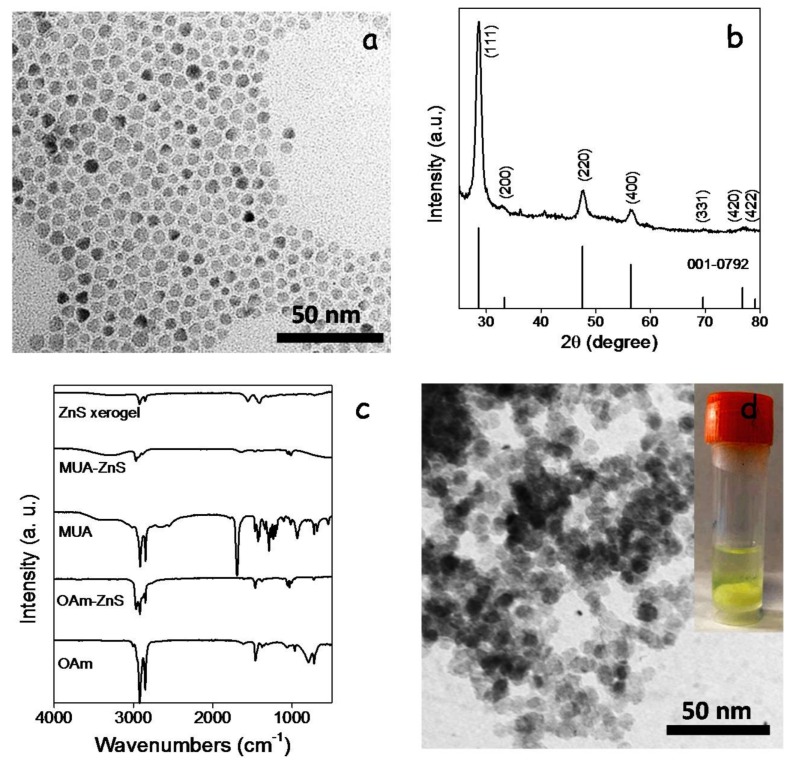
(**a**) TEM micrograph of ZnS NCs; (**b**) XRD pattern of ZnS NCs including the JCPDS 001-0792 phase standard card corresponding to sphalerite ZnS as reference; [21] (**c**) from bottom to top, FTIR spectra of: OAm, as-produced ZnS NCs (OAm-ZnS), MUA, ZnS NCs obtained after ligand exchange with MUA (MUA-ZnS) and ZnS NC-based xerogel; (**d**) TEM micrographs of the ZnS xerogel obtained from MUA-NCs treated with TNM. Inset shows an optical image of the formed ZnS gel.

ZnS NCs were produced following the procedure reported by Hyeon et al. [19], reacting ZnCl_2_ with elemental sulfur in OAm at 320 °C (see details in the experimental section). ZnS NCs produced following this procedure displayed the sphalerite crystal structure and quasi-spherical geometry with an average size of 10 nm (Figure 7a,b). The growth of ZnS NCs and their colloidal stability were controlled by the presence of OAm at their surface as observed by FTIR characterization (Figure 7c) and reported previously [20]. We replaced OAm with MUA by shaking and sonicating a bi-phase solution of OAm-ZnS NCs in hexane and MUA in methanol. Through this process, ZnS NCs moved from the hexane to the methanol phase, where they were stabilized by MUA (MUA-ZnS). Then, the upper hexane solution containing the displaced OAm was discarded and the MUA-ZnS NCs in the methanol solution were collected and further purified as described in the experimental section.

Porous layers of interconnected ZnS NCs were produced following the same procedure as for CGS NCs, using TNM as a non-oxygen transferring agent. Bulk ZnS gels and xerogels were also produced by adding TNM to a colloidal solution of MUA-ZnS NCs in methanol. Figure 7d shows a representative TEM micrograph and an optical image of the interconnected ZnS NC network produced upon addition of TNM to the colloidal solution of MUA-ZnS NCs. FTIR spectra from these gels showed a reduction of the intensity of the peaks at 2924 and 2830 cm^−1^ attributed to organic ligands, consistent with their partial removal (Figure 7c). Notice that, due to the larger size of MUA compared with TGA, the intensity of the peaks associated with C–H vibrations in the FTIR spectrum of the ZnS-based gel is stronger than in the CGS-based one.

**Figure 8:**
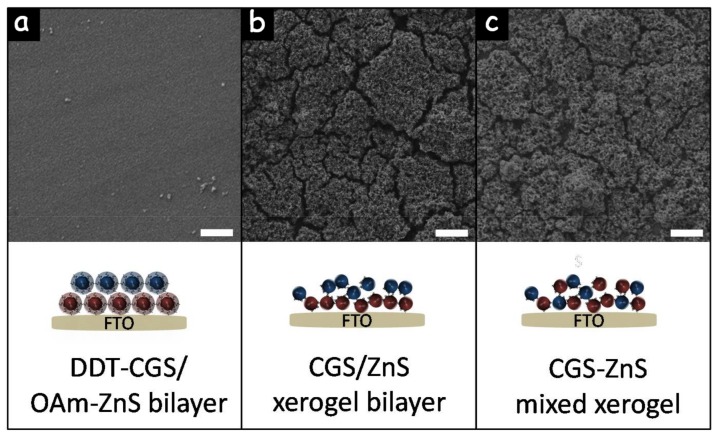
SEM micrographs and schematic representations of the following structures: (**a**) a bilayer obtained from the sequential spin coating of DDT-CGS NCs and OAm-ZnS NCs; (**b**) a bilayer produced from the spin coating and gelation of TGA-CGS NCs and the subsequent deposition and gelation of MUA-ZnS NCs; (**c**) a blended CGS-ZnS NC layer obtained from the spin coating of a solution containing both TGA-CGS NCs and MUA-ZnS NCs followed by treating it with TNM. Scale-bars = 1 μm.

Combining p-type CGS with n-type ZnS in bilayer structures or blended layers should allow a more effective separation of photogenerated charge carriers, reducing charge recombination and promoting photocatalytic performance. Additionally, a faster charge carrier separation should promote the material chemical stability, since metal sulfides suffer from significant photocorrosion during photocatalytic reactions associated with the surface accumulation of photogenerated holes that induce rapid oxidation of lattice S^2−^ ions to S^0^ or soluble sulfates [22,23]. Therefore, beyond single component films, we produced CGS/ZnS bilayers and CGS-ZnS mixed layers. Figure 8a,b show top-view SEM micrographs of CGS/ZnS bilayers produced from the sequential spin-coating of DDT-CGS and OAm-ZnS NCs (Figure 8a) and from the sequential spin coating and gelation of TGA-CGS and MUA-ZnS NCs (Figure 8b). Figure 8c shows a top-view SEM micrograph of the layer produced from the spin coating and subsequent gelation of a solution containing a blend of TGA-CGS and MUA-ZnS NCs. Note that a thermal treatment (250 °C for 60 min under Ar atmosphere) was required to stabilize the DDT-CGS layer before deposition of the OAm-ZnS NCs layer, in order to prevent dissolution of the former. However, such thermal treatment was not necessary for layer deposition on top of gelated materials. This represents a clear advantage over multiple layer deposition procedures that require an annealing step in between each process.

The performance of CGS NC-based layers was evaluated against the photoelectrocatalytic hydrogen evolution reaction in a 0.1 M aqueous solution of Na_2_SO_4_ at neutral pH. Photoelectrocatalytic measurements were carried out with a three-electrode electrochemical cell using the NC-based layers annealed at 250 °C for 60 min as working electrodes.

**Figure 9:**
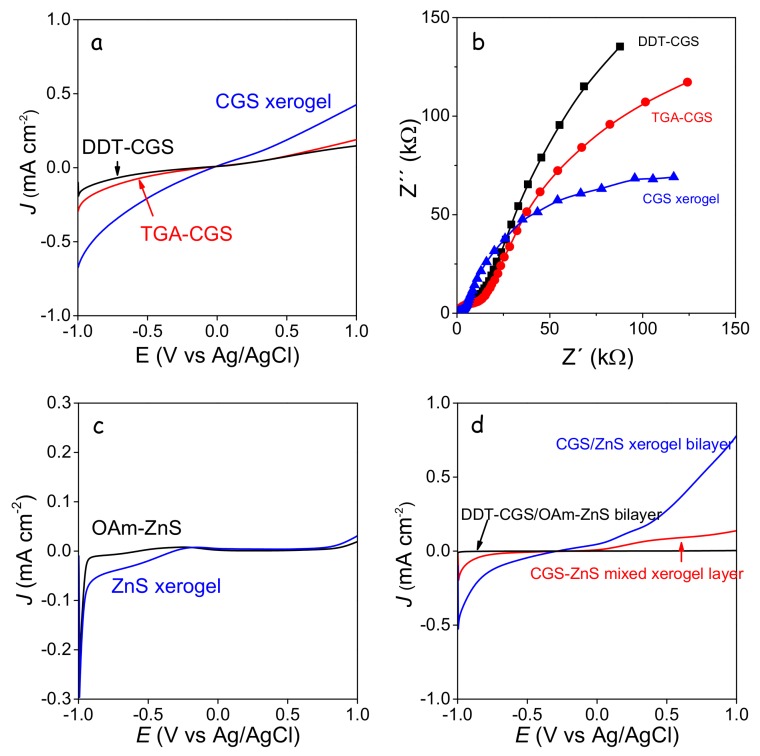
(**a**) linear sweep voltammogram curves at a scan rate of 0.1 V·s^−1^ of DDT-CGS, TGA-CGS and gelated TGA-CGS NC-based layers; (**b**) Nyquist plots for DDT-CGS, TGA-CGS and CGS xerogel films; (**c**) linear sweep voltammogram curves at a scan rate of 0.1 V·s^−1^ of OAm-ZnS and gelated MUA-ZnS NC-based layers; (**d**) linear sweep voltammogram curves at a scan rate of 0.1 V·s^−1^ of DDT-CGS/OAm-ZnS NC-based bilayer, a gelated TGA-CGS/MUA-ZnS NC-based bilayer and a gelated layer produced from a blend of TGA-CGS and MUA-CGS.

Note that no catalyst was used in these experiments, thus overall layer performances were relatively low, but still qualitatively significant to probe dissimilarities between the differently treated samples. Compared with the layers produced from DDT-CGS, higher current densities were obtained from the layers prepared with a shorter organic ligand (TGA-CGS) and particularly from the gelated layer (CGS xerogel) (Figure 9a). This experimental evidence is associated with two properties: (i) the higher surface area of the CGS xerogel, which provided enhanced interaction with the media; and (ii) the enhanced charge carrier transport within the TGA-CGS layer and particularly the CGS xerogel layer compared with DDT-CGS. EIS measurements confirmed the lower impedance of the gelated layers compared with DDT-CGS and TGA-CGS films, associated again with the lower organic content and the higher surface area of the xerogel films (Figure 9b). Similar results were obtained from ZnS layers, demonstrating significantly larger current densities for ZnS xerogels than for OAm-ZnS-based layers (Figure 9c).

Current densities were increased when combining CGS and ZnS xerogels into a bilayer structure, which we associated with a favorable surface energy band arrangement. On the other hand, blended xerogel layers were characterized by lower current densities probably associated to the reduced charge carrier mobility in a nanocrystalline network containing a large density of energy barriers introduced by the random distribution of the p-type and n-type semiconductors.

**Figure 10:**
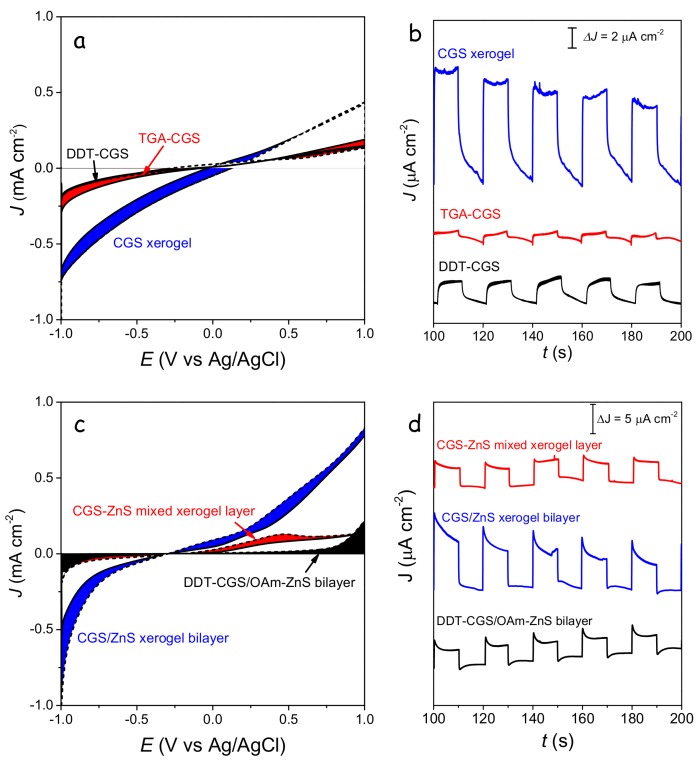
(**a**,**b**) linear sweep voltammogram curves at a scan rate of 0.1 V·s^−1^ (**a**) and time-dependent characteristics (**b**) of the photocurrent response of CGS layers having different surface chemistries. (**c**,**d**) linear sweep voltammogram curves at a scan rate of 0.1 V·s^−1^ (**c**) and time-dependent characteristics (**d**) of the photocurrent response of CGS/ZnS bilayers and a CGS-ZnS mixed layer. Time-dependent characteristics were obtained by applying −0.6 V with respect to an Ag/AgCl reference electrode.

The highest photocurrents were obtained from CGS xerogel monolayers and CGS/ZnS xerogel bilayers (Figure 10). Improved photocatalytic performance with respect to DDT-CGS, TGA-CGS and DDT-CGS/MUA-ZnS layers was related to the enhanced charge transport within the interconnected NC network and the larger surface area of the porous xerogel films. CGS/ZnS xerogel bilayers provided even higher photocurrents than CGS xerogel layers, which we attributed to the more efficient charge separation at the p-n junctions, reducing recombination, and possibly to a more efficient injection of charge to the solution species through a proper band adjustment.

## 4. Conclusions

We demonstrated the formation of porous layers of CGS and ZnS from the treatment of TGA-capped CGS NCs and MUA-capped ZnS NCs with a non-oxygen transferring agent, TNM. This oxidizing agent indirectly created chalcogen-chalcogen bonds between the NCs, anchoring them together. Compared to organic-capped layers, CGS xerogel films were characterized with higher current densities and photoresponses due to improved interparticle coupling and the porous structure. We further produced CGS/ZnS NC-based bilayers and CGS–ZnS NC-based composite layers that exhibited higher current densities and photoresponses than layers deposited from as-produced NCs. In particular, porous CGS/ZnS bilayers showed the highest current densities and photocurrents, associated with improved charge transport due to chalcogen-chalcogen cross-linking between NCs, enhanced interaction with the media due to its high surface area, and more efficient charge separation in the p-n bilayer structure.
